# Classification of White Blood Cells: A Comprehensive Study Using Transfer Learning Based on Convolutional Neural Networks

**DOI:** 10.3390/diagnostics12122903

**Published:** 2022-11-22

**Authors:** Thinam Tamang, Sushish Baral, May Phu Paing

**Affiliations:** 1 Madan Bhandari Memorial College, New Baneshwor, Kathmandu 44600, Nepal; 2 Department of Robotics and AI, School of Engineering, King Mongkut’s Institute of Technology Ladkrabang, Bangkok 10520, Thailand; 3 Department of Biomedical Engineering, School of Engineering, King Mongkut’s Institute of Technology Ladkrabang, Bangkok 10520, Thailand

**Keywords:** complete blood clount, deep learning, label smoothing, mixup augmentation, normalization

## Abstract

White blood cells (WBCs) in the human immune system defend against infection and protect the body from external hazardous objects. They are comprised of neutrophils, eosinophils, basophils, monocytes, and lymphocytes, whereby each accounts for a distinct percentage and performs specific functions. Traditionally, the clinical laboratory procedure for quantifying the specific types of white blood cells is an integral part of a complete blood count (CBC) test, which aids in monitoring the health of people. With the advancements in deep learning, blood film images can be classified in less time and with high accuracy using various algorithms. This paper exploits a number of state-of-the-art deep learning models and their variations based on CNN architecture. A comparative study on model performance based on accuracy, F1-score, recall, precision, number of parameters, and time was conducted, and DenseNet161 was found to demonstrate a superior performance among its counterparts. In addition, advanced optimization techniques such as normalization, mixed-up augmentation, and label smoothing were also employed on DenseNet to further refine its performance.

## 1. Introduction

Blood is a highly specialized tissue that is comprised of several cell types and plasma components. This essential fluid transports and supplies oxygen and nutrition to tissues and organs of the body. Blood functions to remove carbon dioxide, ammonia and other waste products. It also aids in various other biological functions, including oxygen transport, cell regeneration, clotting, body temperature regulations and immunity. There are four essential cellular components in blood: red blood cells (RBCs), white blood cells (WBCs), platelets and plasma. Among them, RBCs typically account for 40–50% of total blood volume and convey oxygen from the lungs to all other vital tissues [1]. On the other hand, WBCs can be found not only in blood but also in lymphatic tissues. Even though they make up a small percentage of blood volume, typically in the range of 1% in a healthy person, they constitute the immune system’s first call against foreign invaders [1]. WBCs look for, detect, and bind to foreign proteins in bacteria, viruses, and fungi so as to eliminate them. There are several types of WBCs, each of which plays a different role in immunity responses [2].

**Figure 1:**
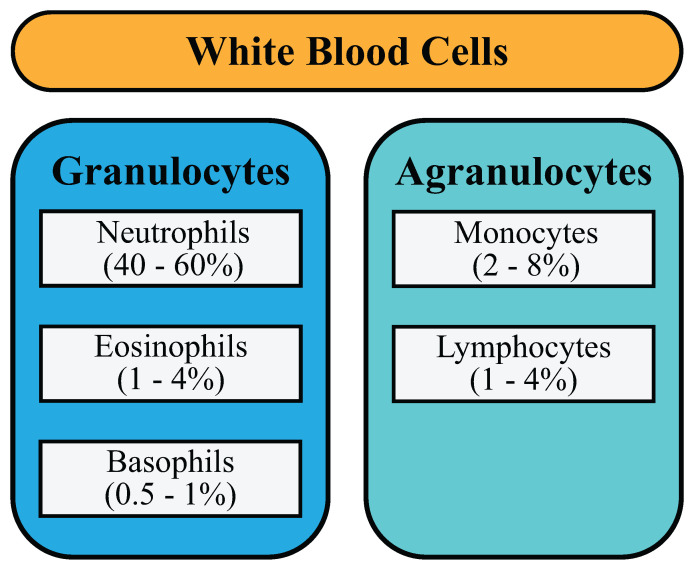
Categorization of White Blood Cells.

CBC (complete blood count) is a widely used blood test to measure a population’s health status. The test entails the determination of RBC, WBC and platelet parameters. Most parameters can be investigated by automatic blood analyzers provided by several manufacturers. The WBC count represents essential parameters on the total WBC, and the differential WBC count displays the absolute number or percentage of neutrophils, eosinophils, basophils, lymphocytes and monocytes. These five categories of WBCs can be categorized as granulocytes and agranulocytes, as depicted in Figure 1. Each WBC type has its own specific function; the alteration of each cell type number reflects the pathological condition of the patient. The results of the WBC differential count are provided by blood analyzers with differential principles based on granularity, size, biochemistry properties, etc. However, blood film examination by skilled and experienced medical technologists is highly needed [3,4]. Traditional methods for blood film examination for counting white blood cells can be imprecise and time-consuming, and the technical skill of laboratory technicians has a substantial impact on the test’s reliability. This paper presents a deep learning approach to perform image categorization to achieve more robust and accurate results. Deep learning approaches similar to this paper are being used in various sectors of medical applications [5,6,7,8].

Although deep learning has become popular in image classification in various medical domains, WBC classification is also a significant task where many variations of different architectures are employed. Even after solving this problem using many algorithms, a comparative analysis of a different model for evaluation and deployment is still lacking. This paper is focused on providing a comparative analysis of various models of CNNs used for the classification of blood smear images. Thus, it is worthwhile to investigate and implement such models for identifying and counting WBCs and providing results based on different validation criteria, nflops, and time complexity, and the best model is suggested. This study is based on the BCCD dataset, and the models used in this study utilize transfer learning techniques and are fine-tuned using the latest advancements that can be specifically customized for image classification tasks. Thus, transfer learning is an important method that facilitates the training of accurate models quickly with fewer data points at a low cost. The methodology applied in this paper uses various steps, i.e., data acquisition, data cleaning, and image processing, followed by the implementation of the models.

Several methods have been developed for categorizing the types of WBCs in blood smear pictures. Most of those methods were based on fuzzy logic, machine learning, deep learning, or a hybrid of these three methods [9,10]. Saraswat et al. [9], in 2014, and Kumar et al. [10], in 2020, surveyed the employment of locally accessible datasets in their majority of research works, building a tailored model for the classification of the data. The distributions of classes in the datasets used in the literature are shown in Figure 1. Most of the studies used an open dataset, whereas other study papers did not divulge their dataset. However, open datasets are preferred for comparison with past publications.

Support-vector machines (SVM) [11] and Bayesian classifiers [12] can be used to classify data using machine learning. Compared to other models, these models can perform well in spite of the large amounts of data with pre-processing in advance. Hegde et al. [13] compared a conventional image classification method and a convolutional neural network (CNN). Although the results are similar, primitive methods rely on image segmentation and feature extraction, but they are easy to implement. In comparison, the CNN is independent of these parameters but requires a large amount of labelled datasets. Singh et al. [14] trained a CNN for 200 epochs and hence proposed a classification model for WBCs. Combining ResNet and DCGAN, Ma et al. [15] came up with a classification model performing better than before. An approach for extracting the region of interest from the smear images for the classification of WBCs was conducted by Sengür et al. [16]. Further developing the concept, Sengür et al. [16] used ResNet50 for the feature extraction and principal component analysis for feature selection, and finally, the classification was performed using long short-term memory. The model performed at 85.7% accuracy. Patil et al. [17] employed canonical correlation analysis [18], enlisting both convolutional and recurrent neural network architectures. The aforementioned researchers [16,17] performed their experiments on the same dataset. Wijesinghe et al. [19] used the K-means clustering method for WBC nuclei segmentation, following which a VGG-16 architecture [20] was employed to classify the designated classes for the nuclei images. A model based on a CNN trained using the local image data proposed by Jung et al. [21] was used for classifying the data from another reference presented in [22].

Ucar et al. [23] suggested a ShuffleNet [24]-based deep learning model, producing an overall accuracy of 97 percent. Using Euler’s Jenks optimisation, Karthikeyan et al. [25] devised a CNN for detecting and classifying WBC. Using Jenks optimized pooling [22] in the blood samples, peripheral RBCs were removed. Moreover, for learning-based approaches, research based on fuzzy logic can also be found in the literature. Ghosh et al. [26] presented a fuzzy technique for counting WBCs in smear images. Similarly, a technique used by Chan-Vese [27] was utilized by Rawat et al. [28] to detach WBC nuclei from sample images; they also proposed an adaptive neuro-fuzzy classifier. Recently, Ashish et al. proposed the SOTA model based on CNN architecture for the classification of WBCs in fewer epochs, i.e., in a more time-efficient m anner, and achieved an accuracy of 98.55%.

This paper utilizes a convolutional neural network (CNN) [29] and its variants, such as AlexNet [30], DenseNet-(121, 161) [31], ResNet-(18, 34, 50) [32], SqueezeNet-(10, 11) [33], and VGGNet-(11, 13) [20], for the classification of white blood cells from smear images of blood gathered on the Blood Cell Count and Detection dataset. Different architectures of CNNs have been experimented with, and the results are portrayed and validated using the validation criteria provided in the following Section 2.6.

The core concepts and contributions of this paper are as follows:

1. We have applied advanced image processing and data augmentation techniques, i.e., random resizing and cropping, which randomly select different parts of an image, enabling the model to focus on and perceive various features. This improves the model’s generalization capabilities and prevents overfitting.
2. We applied advanced fine-tuning techniques such as normalization, mixup augmentation, and label smoothing to train the CNN model and obtain preferable results in comparison with other similar research.
3. We investigated and compared the efficiency and complexity of multiple deep neural network (DNN) architectures initialized with pre-trained weights for WBC classification.

## 2. Materials and Methods

### 2.1. Dataset

The BCCD dataset [34] is an open dataset containing five classes of white blood cell images. The dataset is comprised of 12,436 images of blood cells managed into five different categories, i.e., basophils, eosinophils, lymphocytes, monocytes, and neutrophils. Out of the five categories, basophils are removed during data clean-up due to a lack of image availability. The instance distribution on the remaining four classes has approximately 300 images per class.

### 2.2. Data Pre-Processing

**Figure 2:**
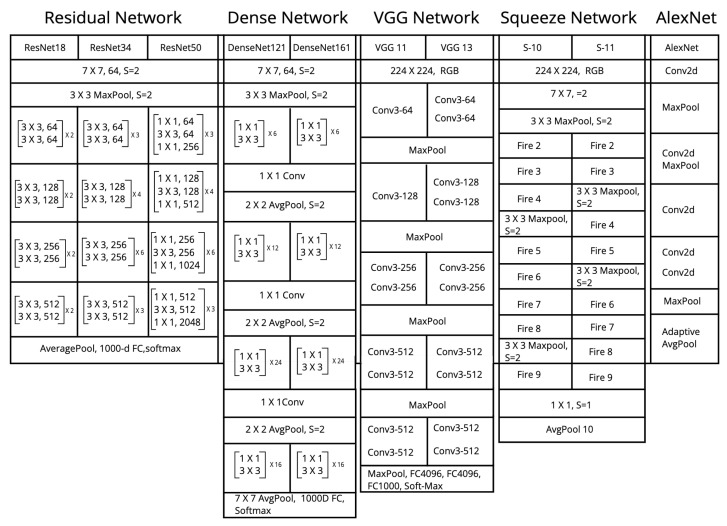
Models used in the experimentation of WBC classification.

After data acquisition, the data were thoroughly scrutinized, where it was discovered that the class basophil has a meagre image count; hence, the field was removed entirely before feeding the data to the pre-trained CNN models, as listed in Figure 2. Certain pre-processing of the data was carried out. We performed three operations within the datablock, as mentioned below:

- Data split: This helps in folding the dataset into train, test and validation sets.
- Random resize: This block resizes our data to create uniformity in the image size.
- Data augmentation: Image transformations are performed during the run time using this block.

### 2.3. Convolutional Neural Network

In one of the multidisciplinary fields of AI, deep learning, convolutional neural networks are considered one of the advanced architectures for various computer vision tasks. In comparison to other networks, CNNs have demonstrated higher achievements in computer vision [35].

CNNs have a specific trait called invariance that allows them to see images in a very broad fashion [36] so that even an image with scattered face attributes is treated as a person by CNNs. Convolution is a feature extraction procedure in a CNN that employs a kernel of a specific size. The kernel is traversed throughout the network with specific steps, i.e., stride, which is specified during the implementation of the architecture. The outcome of this technique is known as a feature map. Following the extraction of the feature map, the pooling procedure is used to reduce the size of the feature map even more [29]. A layer in the network is made up of the processes outlined above. The network is made up of numerous layers. The image is finally flattened, and a fully or partially connected layer is formed [29]. The image is then classified using a classification layer, which determines the likelihood of the image falling into one of several categories.

In this paper, the different architectures of CNN are used as listed in Figure 2, which has been further fine-tuned to create models that are specifically tailored for WBC image classification.

### 2.4. Transfer Learning

Transfer learning is a technique that enables researchers and practitioners to employ a previously learned model for a completely new task. In computer vision, transfer learning can be beneficial in leveraging knowledge from a previous assignment to improve the prediction of a new task. Due to these capabilities and the ability to train a deep network with few inputs, this technique is gaining more attention in the field.

Transfer learning only functions if the skills acquired in the first task are generic. In addition, the input to the model must be of the same size as when it was first trained. In this case, we must perform a resizing operation before feeding it to the network.

**Figure 3:**
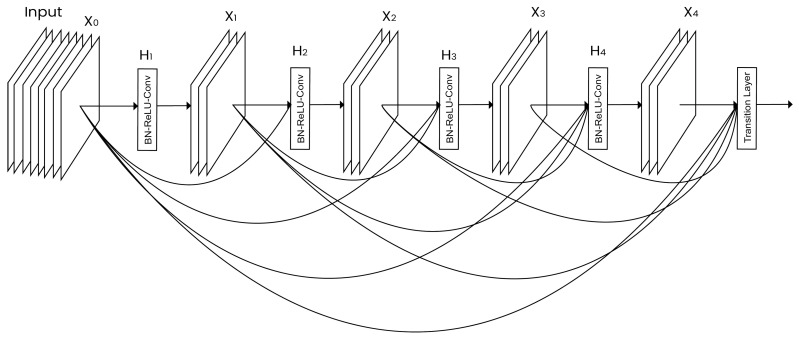
Dense convolutional network.

- DenseNet: Introduced by Huang et al. [31], this network contains direct connections between any two layers having the same size as the feature maps. DenseNet, as shown in Figure 3, reduces the vanishing gradient problem, reinforces the feature propagation, vitalizes the reuse of features, and significantly decreases the number of parameters.
- ResNet: Introduced by He et al. in 2015 [32]. It demonstrates the use of residual modules to show that the standard SGD and an equitable initialization function can be used for training deep networks. Furthermore, residual modules were updated to use identity mappings in 2016 [37].
- AlexNet: Introduced by Krizhevsky et al. [30]. The architecture is built using eight layers, where the first five are used for the convolution, and the remaining layers are fully connected layers with a softmax function in the last layer.
- SqueezeNet: Introduced by Iandola et al. in 2016 [33]. SqueezeNet begins with a convolutional layer, followed by nine fire modules, and ends with a convolutional layer. The fire modules in the architecture contain a max pooling operation, where the max pooling has a stride of 2.
- VGGNet: This architecture was introduced by Simonyan and Zisserman in 2014 [20]. Only 3 × 3 convolutional layers are used in the VGG network, and they are stacked on top of one another in increasing depths. In addition, max pooling handles reduce volume size. Next comes a softmax classifier, which is composed of two completely connected layers.

### 2.5. Model Building

Models were run for a total of 9 epochs, where the first 3 epochs did not perform any training of the network (i.e., freeze). In the next 6 epochs, we trained the network and observed the performance (i.e., unfreeze). After training, we used the validation set to test the model’s performance. Therefore, the methodology is carried out for all the pre-trained models mentioned earlier, and the most efficient model out of them, i.e., DenseNet-161, is selected as our main model. Since DenseNet161 outperforms other models, the paper shows the most experimentation results obtained from DenseNet-161.

### 2.6. Performance Evaluation Metrics

The entire performance of a classifier is not shown by accuracy alone. As a result, several performance indicators such as precision, recall, F1-score, and ROCAUC were computed [38]. The ratio of true predictions among all the predictions generated by any classifier for a given class is known as precision. Recall or sensitivity, on the other hand, refers to the proportion of accurate predictions for a class out of all the samples in that class. A statistic known as the F1-score combines precision and recall into one metric for assessing the performance of a classifier. There are mathematical definitions for each of these performance indicators, which are presented below. The mathematical illustrations have the following acronyms for true positives (TP), false positives (FP), true negatives (TN), and false negatives (FN).

(1) $$Precision=\left(\frac{TP}{TP+FP}\right)$$

(2) $$Recall=\left(\frac{TP}{TP+FN}\right)$$

(3) $$F1-score=\left(2*\frac{Precision*Recall}{Precision+recall}\right)$$

The area under the curve (AUC) of an ROC curve is a performance statistic for classification issues at multiple threshold levels. An ROC is a probability curve, whereas the AUC is the level or measure of separability. This can be considered as an indicator to depict how well any model is able to distinguish between classes.

The AUC shows how accurately the model predicts that the 0 class will be 0 and that the 1 class will be 1. If the model predicts a 0 class as 0 and a 1 class as 1 better, the value for the AUC will be higher, indicating the model is better at classification.

### 2.7. Hardware

Experiments were performed in a Google Colaboratory environment using the provided GPU Tesla T4, 12 GB RAM and Intel Xenon CPU 2.20 GHz. Python libraries used for this experimentation included FastAI, PyTorch and NumPy libraries.

## 3. Results and Discussion

**Figure 4:**
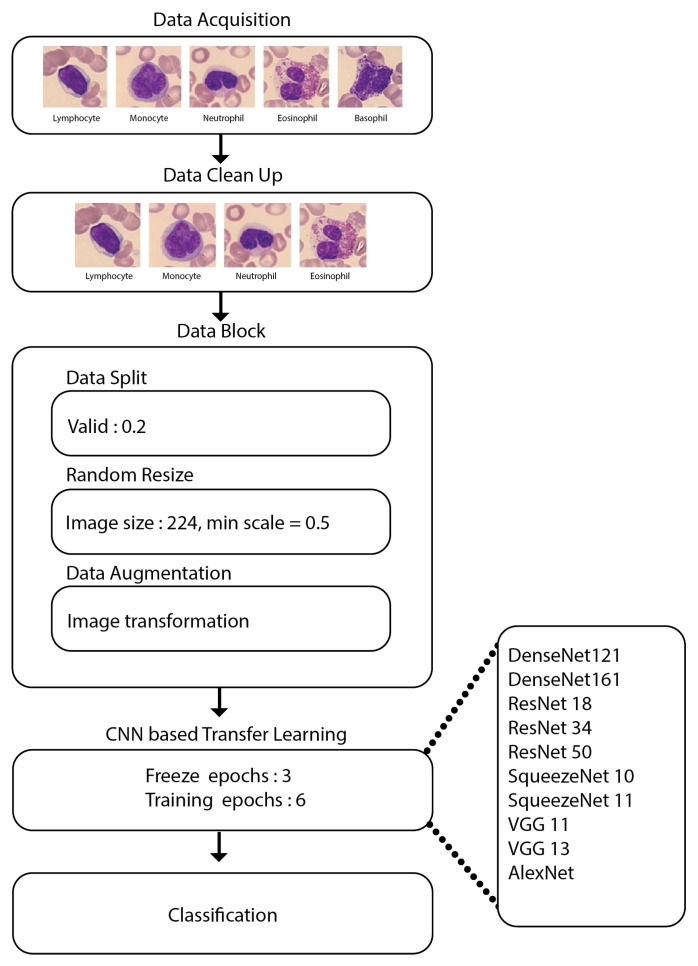
Methodology.

This research was carried out using the methodology shown in Figure 4.

The proposed classification approach’s effectiveness was validated on the publicly accessible (BCCD) dataset.

### 3.1. Experimental Details

The raw image dataset from BCCD underwent several pre-processing steps, as listed below.

1. Data wrangling: The dataset contains only three images of the basophil class, since it may show little significance when compared to other forms of WBCs. As a result, this form of WBC was removed, and the CNN models were trained with the four different types of WBCs.
2. Constructing a datablock: In Fastai [39], a datablock is a high-level application programming interface. It is a method for carefully defining all of the stages involved in preparing data for deep learning systems. The fundamental components of the datablock are listed below:
  (a) Train-test division: The splitting ratio was 8:2 for dividing the dataset into training and testing.
  (b) Resizing of images: Every image from the dataset was converted to 224 × 224 size, and then the entire batch of images was converted to 128 × 128.
  (c) Data augmentation: The resized images underwent further data augmentation operations, i.e., rotation, zoom, perspective warping, and lighting (change in brightness and contrast). This process of generating random variations of the input data so that they appear unique but do not alter the data’s underlying significance is referred to as data augmentation.
    Data augmentation is the process of generating random variations of the input data so that they appear unique but do not alter the data’s underlying significance.
  (d) Model hyperparameters: The settings used for model building were as follows:
    - Learning rate: 0.001;
    - Optimizer: Adam;
    - Epochs: 9;
    - Batch size: 64;
    - Loss function: cross-entropy loss function.
3. Dataloaders: They store multiple dataloader objects (i.e., a training dataloader and a validation dataloader).

### 3.2. Model Benchmarking

In this paper, for the classification of the WBC images, different variants of the CNN were implemented. Their performance was tested using the BCCD dataset; hence, the results obtained are compared in this section. It is observed from the experiments that the results improve as the network gets deeper. Here, in this case, the deepest network of all has the best value. To be precise, the accuracy obtained during both training and testing is 100%. Table 1 depicts the parameters necessary to compare the models: average time, trainable parameters and accuracy. Every model with its average time taken while processing the trainable parameters with some accuracy is well illustrated. Apart from the table, this paper also shows the graph of the training and testing process of DenseNet-161. DenseNet-161 is the best-performing network among the 10 networks used in the comparison of networks. The validation report of DenseNet-161 is also provided.

### 3.3. Model Behavior

#### 3.3.1. Normalization

Normalization, which was first introduced by Ioffe and Szgedy et al. [40] in 2015, was used for normalizing the output from the activation in each layer before the signal traversed to the next layer.

Let us consider *x* as a mini-batch of activation; then, the normalized $\hat{x}$ can be obtained using the equations below:

(4) $${\hat{x}}_{i}=\left(\frac{x_{i}-\mu_{\beta }}{\sqrt{\sigma_{\beta }^{2}+\epsilon }}\right)$$

(5) $$\mu_{\beta }=\left(\frac{1}{M}\sum_{i=1}^{m}x_{i}\right)$$

(6) $$\sigma_{\beta }^{2}=\left(\frac{1}{M}\sum_{i=1}^{m}{\left(x_{i}-\mu_{\beta }\right)}^{2}\right)$$

We calculate the $\mu_{\beta }$ and $\sigma_{\beta }$ over each mini-batch β during training. Applying this equation suggests that the activations will be zero-centered, with a mean and variance that are both close to zero. We substitute the mini-batch $\mu_{\beta }$ and $\sigma_{\beta }$ with running averages of $\mu_{\beta }$ and $\sigma_{\beta }$, which are calculated during the training phase at testing time. This guarantees that we can process images through our network and still obtain correct predictions free from bias caused by the $\mu_{\beta }$ and $\sigma_{\beta }$ from the final mini-batch processed through the network during training.

The comparison of different performance metrics in DenseNet-161 after normalization is given in Table 2.

#### 3.3.2. Mixup Augmentation

Mixup, which was introduced by Hongyi Zhang et al. [41], is a potent data augmentation strategy that can offer significantly improved accuracy, especially when one doesn’t have much data or a pre-trained model that was trained on data that are similar to one’s dataset. For each image, the following are the steps performed by mixup:

- Pick another data at random from your dataset;
- Randomly choose a weight;
- Take a weighted average of your image and the image you choose in step 2; this will serve as your independent variable;
- Take a weighted average of the labels on this image and the labels on your image; the result will be your dependent variable.

$x_{i}$ and $x_{j}$ are raw input vectors, whereas $y_{i}$ and $y_{j}$ are one-hot label encodings, and λ is the weight for our weighted average. The idea of mixup augmentation is described in the equations below:

(7) $$\tilde{x}=\lambda x_{i}+\left(1-\lambda \right)x_{j};\tilde{y}=\lambda y_{i}+\left(1-\lambda \right)y_{j}$$

The comparison of different performance metrics in DenseNet-161 after mixup augmentation is given in Table 2.

#### 3.3.3. Label Smoothing

Label smoothing was introduced by Christian Szegedy et al. [42]. In order to save memory, generally in classification problems, we avoid one-hot encoding in practice, but the loss we compute is the same as if we had. This indicates that the model has been trained to return 0 for all categories other than the one that belongs to the target class, which returns 1. The model will acquire gradients and develop the ability to forecast activations with even greater confidence if 0.999 is deemed to be “good enough.” This promotes overfitting and results in a model that, at the moment of inference, will not provide helpful probabilities: it will always report 1 for the predicted category, even if it is not entirely sure, simply because this is how it was trained.

Instead, we could train by replacing all 1s with numbers just below 1 and all 0s with numbers a little above 0, respectively. Label smoothing is the term for this. Label smoothing will help make the training more robust even if there is mislabeled data by encouraging models to be less confident. A model that generalizes more effectively will be the end outcome.

In practice, we start with one-hot-encoded labels, then replace all 0s with $\left(\frac{\epsilon }{N}\right)$, where *N* is the number of classes and ϵ is a parameter. Similarly, we replace the 1s with $\left(1-\epsilon +\frac{\epsilon }{N}\right)$. In this way, we prevent the model from making overly confident predictions.

The comparison of different performance metrics in DenseNet-161 after label smoothing is given in Table 2.

## 4. Conclusions

Manual classification of blood cells visually by experts is a time-consuming and tiresome endeavour. The presented method described herein has successfully been demonstrated to be capable of classifying the types of white blood cells by combining smear images with appropriate deep learning approaches. The white blood cell categorization task for leukocytes can be completed automatically based on the results of the proposed approach.

In this study, we looked at how to classify smear images via the use of several CNN architectures. On the basis of trainable parameters, the average time taken and accuracy, the best results obtained after implementing the discussed models were compared to each other. In comparison to other architectures, DenseNet-161 performed substantially better in the leukocyte recognition challenge, with significantly higher accuracy. However, the superiority of the accuracy of DenseNet-161 is challenged by the other implemented models when additional variables such as average time taken and the number of trainable parameters are taken into account. From this paper, we gained insights into how multiple parameters need to be taken into account for selecting a suitable deep learning architecture. In addition, various architectures and networks can be utilized for benchmarking models, while larger datasets may give rise to different results.

## Figures and Tables

**Table 1:** Performance analysis of various models.

Model	Average Time	Trainable Parameters	Accuracy
AlexNet	0:52	2,735,936	0.9702
DenseNet 121	2:89	8,009,600	0.9967
DenseNet 161	4:24	28,744,896	1.0
ResNet 18	1:10	11,705,920	0.9939
ResNet 34	1:44	21,814,080	0.9979
ResNet 50	2:52	25,616,448	0.9991
SqueezeNet 10	1:01	1,264,832	0.9851
SqueezeNet 11	0:57	1,251,904	0.9754
VGG Net 11	2:24	9,755,392	0.9939
VGG Net 13	3:40	9,940,288	0.9967

**Table 2:** Summary of model performance.

Epoch	Training Loss	Validation Loss	Accuracy	Error Rate	F1	Precision	Recall	ROCAUC	Time
Normalization									
0	0.140218	0.057371	0.978689	0.021311	0.978645	0.978585	0.979020	0.999439	04:24
1	0.100621	0.014288	0.996783	0.003217	0.996785	0.996747	0.996825	0.999965	04:23
2	0.055214	0.010322	0.996381	0.003619	0.996385	0.996256	0.996565	1.000000	04:24
3	0.040718	0.002779	0.999196	0.000804	0.999196	0.999239	0.999155	1.000000	04:23
4	0.022699	0.001568	1.000000	0.000000	1.000000	1.000000	1.000000	1.000000	04:23
5	0.017631	0.001189	1.000000	0.000000	1.000000	1.000000	1.000000	1.000000	04:23
Mixup Augmentation									
0	0.632420	0.089475	0.978287	0.021713	0.978325	0.978680	0.978054	0.999194	04:29
1	0.555610	0.072873	0.983514	0.016486	0.983525	0.983634	0.984351	0.999898	04:32
2	0.503748	0.042065	0.995979	0.004021	0.995983	0.995864	0.996143	0.999975	04:32
3	0.477047	0.033872	0.999196	0.000804	0.999196	0.999158	0.999237	0.999999	04:29
4	0.452092	0.028028	0.999196	0.000804	0.999196	0.999196	0.999196	0.999999	04:30
5	0.444563	0.027773	0.999196	0.000804	0.999196	0.999196	0.999196	1.000000	04:29
Label Smoothing									
0	0.520124	0.432285	0.979091	0.020909	0.979164	0.979401	0.979894	0.999494	04:29
1	0.442535	0.383923	0.999598	0.000402	0.999598	0.999619	0.999578	0.999997	04:31
2	0.411049	0.366136	0.999598	0.000402	0.999598	0.999578	0.999618	1.000000	04:30
3	0.389663	0.360205	1.000000	0.000000	1.000000	1.000000	1.000000	1.000000	04:29
4	0.379095	0.357764	1.000000	0.000000	1.000000	1.000000	1.000000	1.000000	04:28
5	0.374234	0.356941	1.000000	0.000000	1.000000	1.000000	1.000000	1.000000	04:29

## Data Availability

The data presented in this study are available at the following link: https://www.kaggle.com/datasets/paultimothymooney/blood-cells (accessed on 5 August 2022).

## References

[1] Brundha M., Pathmashri V., Sundari S. (2019). Quantitative Changes of Red Blood cells in Cancer Patients under Palliative Radiotherapy-A Retrospective Study. Res. J. Pharm. Technol..

[2] Aliko V., Qirjo M., Sula E., Morina V., Faggio C. (2018). Antioxidant defense system, immune response and erythron profile modulation in gold fish, *Carassius auratus*, after acute manganese treatment. Fish Shellfish. Immunol..

[3] Seckin B., Ates M.C., Kirbas A., Yesilyurt H. (2018). Usefulness of hematological parameters for differential diagnosis of endometriomas in adolescents/young adults and older women. Int. J. Adolesc. Med. Health.

[4] Dai W.C., Zhang H.W., Yu J., Xu H.J., Chen H., Luo S.P., Zhang H., Liang L.H., Wu X.L., Lei Y. (2020). CT imaging and differential diagnosis of COVID-19. Can. Assoc. Radiol. J..

[5] Vaitkeviciene G., Heyman M., Jonsson O., Lausen B., Harila-Saari A., Stenmarker M., Taskinen M., Zvirblis T., Åsberg A., Groth-Pedersen L. (2013). Early morbidity and mortality in childhood acute lymphoblastic leukemia with very high white blood cell count. Leukemia.

[6] Hu Z., Tang J., Wang Z., Zhang K., Zhang L., Sun Q. (2018). Deep learning for image-based cancer detection and diagnosis-A survey. Pattern Recognit..

[7] Shen L., Margolies L.R., Rothstein J.H., Fluder E., McBride R., Sieh W. (2019). Deep learning to improve breast cancer detection on screening mammography. Sci. Rep..

[8] Mambou S.J., Maresova P., Krejcar O., Selamat A., Kuca K. (2018). Breast cancer detection using infrared thermal imaging and a deep learning model. Sensors.

[9] Saraswat M., Arya K. (2014). Automated microscopic image analysis for leukocytes identification: A survey. Micron.

[10] Anilkumar K., Manoj V., Sagi T. (2020). A survey on image segmentation of blood and bone marrow smear images with emphasis to automated detection of Leukemia. Biocybern. Biomed. Eng..

[11] Zheng X., Wang Y., Wang G., Liu J. (2018). Fast and robust segmentation of white blood cell images by self-supervised learning. Micron.

[12] Sabino D.M.U., da Fontoura Costa L., Rizzatti E.G., Zago M.A. (2004). A texture approach to leukocyte recognition. Real-Time Imaging.

[13] Hegde R.B., Prasad K., Hebbar H., Singh B.M.K. (2019). Comparison of traditional image processing and deep learning approaches for classification of white blood cells in peripheral blood smear images. Biocybern. Biomed. Eng..

[14] Singh I., Singh N.P., Singh H., Bawankar S., Ngom A. (2020). Blood cell types classification using CNN. Proceedings of the International Work-Conference on Bioinformatics and Biomedical Engineering.

[15] Ma L., Shuai R., Ran X., Liu W., Ye C. (2020). Combining DC-GAN with ResNet for blood cell image classification. Med. Biol. Eng. Comput..

[16] Şengür A., Akbulut Y., Budak Ü., Cömert Z. (2019). White blood cell classification based on shape and deep features. Proceedings of the 2019 International Artificial Intelligence and Data Processing Symposium (IDAP).

[17] Patil A., Patil M., Birajdar G. (2021). White blood cells image classification using deep learning with canonical correlation analysis. IRBM.

[18] Hotelling H. (1992). Relations between two sets of variates. Breakthroughs in Statistics.

[19] Wijesinghe C.B., Wickramarachchi D.N., Kalupahana I.N., Lokesha R., Silva I.D., Nanayakkara N.D. (2020). Fully Automated Detection and Classification of White Blood Cells. Proceedings of the 2020 42nd Annual International Conference of the IEEE Engineering in Medicine & Biology Society (EMBC).

[20] Simonyan K., Zisserman A. (2014). Very deep convolutional networks for large-scale image recognition. arXiv.

[21] Jung C., Abuhamad M., Alikhanov J., Mohaisen A., Han K., Nyang D. (2019). W-net: A CNN-based architecture for white blood cells image classification. arXiv.

[22] Rezatofighi S.H., Soltanian-Zadeh H. (2011). Automatic recognition of five types of white blood cells in peripheral blood. Comput. Med. Imaging Graph..

[23] Ucar F. (2020). Deep Learning Approach to Cell Classification in Human Peripheral Blood. Proceedings of the 2020 5th International Conference on Computer Science and Engineering (UBMK).

[24] Zhang X., Zhou X., Lin M., Sun J. Shufflenet: An extremely efficient convolutional neural network for mobile devices. Proceedings of the IEEE Conference on Computer Vision and Pattern Recognition.

[25] Karthikeyan M., Venkatesan R., Vijayakumar V., Ravi L., Subramaniyaswamy V. (2020). White blood cell detection and classification using Euler’s Jenks optimized multinomial logistic neural networks. J. Intell. Fuzzy Syst..

[26] Ghosh P., Bhattacharjee D., Nasipuri M. (2016). Blood smear analyzer for white blood cell counting: A hybrid microscopic image analyzing technique. Appl. Soft Comput..

[27] Chan T.F., Vese L.A. (2001). Active contours without edges. IEEE Trans. Image Process..

[28] Rawat J., Singh A., Bhadauria H., Virmani J., Devgun J. (2018). Leukocyte classification using adaptive neuro-fuzzy inference system in microscopic blood images. Arab. J. Sci. Eng..

[29] Albawi S., Mohammed T.A., Al-Zawi S. (2017). Understanding of a convolutional neural network. Proceedings of the 2017 International Conference on Engineering and Technology (ICET).

[30] Krizhevsky A., Sutskever I., Hinton G.E. (2012). ImageNet classification with deep convolutional neural networks. Adv. Neural Inf. Process. Syst..

[31] Huang G., Liu Z., Van Der Maaten L., Weinberger K.Q. Densely connected convolutional networks. Proceedings of the IEEE Conference on Computer Vision and Pattern Recognition.

[32] He K., Zhang X., Ren S., Sun J. Deep residual learning for image recognition. Proceedings of the IEEE Conference on Computer Vision and Pattern Recognition.

[33] Iandola F.N., Han S., Moskewicz M.W., Ashraf K., Dally W.J., Keutzer K. (2016). SqueezeNet: AlexNet-level accuracy with 50x fewer parameters and <0.5 MB model size. arXiv.

[34] Mooney P. (2018). Blood Cell Images. www.kaggle.com/datasets/paultimothymooney/blood-cells.

[35] Valueva M.V., Nagornov N., Lyakhov P.A., Valuev G.V., Chervyakov N.I. (2020). Application of the residue number system to reduce hardware costs of the convolutional neural network implementation. Math. Comput. Simul..

[36] Sabour S., Frosst N., Hinton G.E. (2017). Dynamic routing between capsules. arXiv.

[37] He K., Zhang X., Ren S., Sun J. (2016). Identity mappings in deep residual networks. Proceedings of the European Conference on Computer Vision.

[38] Olson D.L., Delen D. (2008). Performance evaluation for predictive modeling. Advanced Data Mining Techniques.

[39] Howard J., Gugger S. (2020). Fastai: A layered API for deep learning. Information.

[40] Ioffe S., Szegedy C. Batch normalization: Accelerating deep network training by reducing internal covariate shift. Proceedings of the International Conference on Machine Learning, PMLR.

[41] Zhang H., Cisse M., Dauphin Y.N., Lopez-Paz D. (2017). mixup: Beyond empirical risk minimization. arXiv.

[42] Szegedy C., Vanhoucke V., Ioffe S., Shlens J., Wojna Z. Rethinking the inception architecture for computer vision. Proceedings of the IEEE Conference on Computer Vision and Pattern Recognition.

